# Varicella zoster virus and cytomegalovirus coinfection in a live related kidney transplant recipient: A case report

**DOI:** 10.1002/ccr3.9089

**Published:** 2024-06-16

**Authors:** Amit Bari, Nura Afza Salma Begum, Farnaz Nobi, Harun Ur Rashid, Niyoti Akhter, Sumona Islam

**Affiliations:** ^1^ Department of Nephrology Kidney Foundation Hospital and Research Institute Dhaka Bangladesh; ^2^ Research Wing Kidney Foundation Hospital and Research Institute Dhaka Bangladesh; ^3^ Department of Gastroenterology Delta Medical College and Hospital Dhaka Bangladesh

**Keywords:** coinfection, cytomegalovirus, kidney transplant, live related, varicella zoster virus

## Abstract

**Key Clinical Message:**

The immunomodulatory effect of CMV makes coinfection with other microbes, like VZV possible and potentially deadlier in the post kidney transplant period. Treatment should be started promptly. Both infections can be treated with Valganciclovir.

**Abstract:**

Infections are common complications in kidney transplant recipients owing to the lifelong immunosuppression. Cytomegalovirus (CMV) and Varicella Zoster Virus (VZV) infections are quite common in the posttransplant period. Coinfection with both however has been reported only once. The immunomodulatory effect of CMV makes their interaction with other organisms like VZV potentially sinister. This is a case of a young woman who developed coinfection with HZV and CMV in the first month following a live related kidney transplantation from her mother. Transplant surgery went well with good urine output, but serum creatinine did not fall below 1.7 mg/dL. Immunosuppression consisted of intravenous (IV), followed by oral prednisolone, Mycophenolate Sodium (MPS) and Tacrolimus. 25 days after an uneventful surgery, she developed fever, followed by pain and vesicular eruption on the forehead, typical of VZV infection, along with rising creatinine. CMV PCR yielded 300 copies/mL of DNA, which was undetectable in both donor and recipient pre‐transplant. Total white blood cell count fell to 2 × 10^9^/L. MPS was temporarily stopped. Treatment with Valgancyclovir led to resolution of fever, skin lesions and brought serum creatinine down to baseline over 2 weeks.

## Graphical abstract

Timeline of important events in the peri‐transplant period.

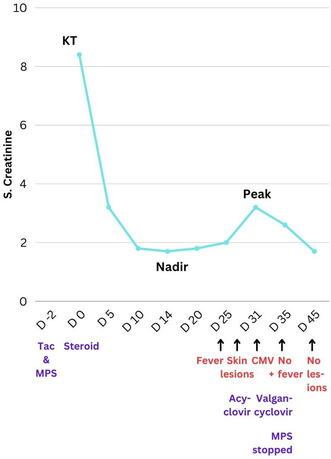

## 1 INTRODUCTION

Kidney transplant is the best long‐term treatment option for end stage kidney disease (ESKD) patients. It restores the entire spectrum of kidney function and provides a better quality of life compared to any other modality of renal replacement therapy (RRT). But this comes at the cost of lifelong immunosuppressive therapy in order to ensure graft survival. Intensive immunosuppression is usually limited to the first year after kidney transplantation, making infection the commonest cause of death in this period.^1^ Although infection and infection related mortality rates are lower in kidney transplant recipients compared to other solid organ transplants, it remains a significant source of mortality and morbidity.

Infection pattern and organisms vary according to time passed after transplant. Opportunistic infections, especially viral, predominate in the first 6 months.^2^ Organisms from the Herpesviridae family are some of the common pathogens. Varicella Zoster virus (VZV) and Cytomegalovirus (CMV) are both members of the Herpesviridae. Compared to other solid organ transplants, CMV infection occurs less frequently following kidney transplants. VZV infection, however occurs more frequently.^3^ The infection rates of CMV and VZV ranges from 8% to 32% and 4% to 12% across studies.^4^ Simultaneous infections with multiple viruses are much less common. There have been few cases of CMV, BK Virus and Human Herpes Virus (HHV) 6 and 7 coinfection.^5^, ^6^ But there has been only one reported case of CMV nephritis and VZV coinfection to our knowledge.^7^

Here we report a case of a VZV and CMV coinfection in a 32‐year‐old female in her first month following a live related kidney transplantation.

## 2 CASE HISTORY/EXAMINATION

A 32‐year‐old ESKD woman underwent live‐related kidney transplantation from her mother following a period of hemodialysis via arteriovenous fistula for 8 months. Although renal biopsy was not done due to her advanced presentation, the primary diagnosis was presumed to be glomerulonephritis based on proteinuria and bilateral symmetrically contracted kidneys. There was one haplotype match and both B and T cell cross matches were negative. CMV DNA was undetected in both donor and recipient prior to transplantation.

The donor being her mother and considering the financial condition of the patient, no depleting or non‐depleting induction therapy was given. According to the institutional protocol, immunosuppression consisted of intravenous (IV) Methylprednisolone 500, 250, and 125 mg on three consecutive days starting on the day of transplant surgery, followed by oral prednisolone 20 mg/day. This was accompanied by oral Mycophenolate Sodium (MPS) 1080 mg/day and Tacrolimus 0.1 mg/kg/day, which was started 2 days before the transplant. During pre‐transplant evaluation, she was found to be anti‐HCV positive. Liver function tests were normal and HCV RNA was undetected. Therefore, a combination of Sofusbuvir and Velpatasvir was also started immediately prior to transplantation at a prophylactic dose of 400/100 mg/day and continued. CMV or Pneumocystis carinii (PCP) prophylaxis was not started.

The surgery was uneventful. Donor had dual renal arteries and single vein of normal course and caliber. The warm ischemic time (WIT) was 37 s and the cold ischemic time (CIT) was 58 min 25 s. The patient had good urine output following the surgery ranging from 4 to 6 L/day, recovered well clinically and did not need any dialysis. However, the kidney function did not become normal following the surgery, reaching a nadir of 2 mg/dL on the 13th postoperative day (POD) and rising after that. Tacrolimus trough level was 8.1 ng/mL, which was within the target range of 8–12 ng/mL, making Tacrolimus toxicity unlikely. She did not consent to a biopsy. Three consecutive doses of 500 mg IV Methylprednisolone were given, suspecting acute rejection, which brought the serum creatinine down to a baseline of 1.7 mg/dL. CMV or *Pneumocystis carinii* (PCP) prophylaxis, which is usually started upon renal function becoming normal or near normal according to institutional protocol was held up to this point.

## 3 METHODS

### 3.1 Differential diagnosis and investigations

**FIGURE 1:**
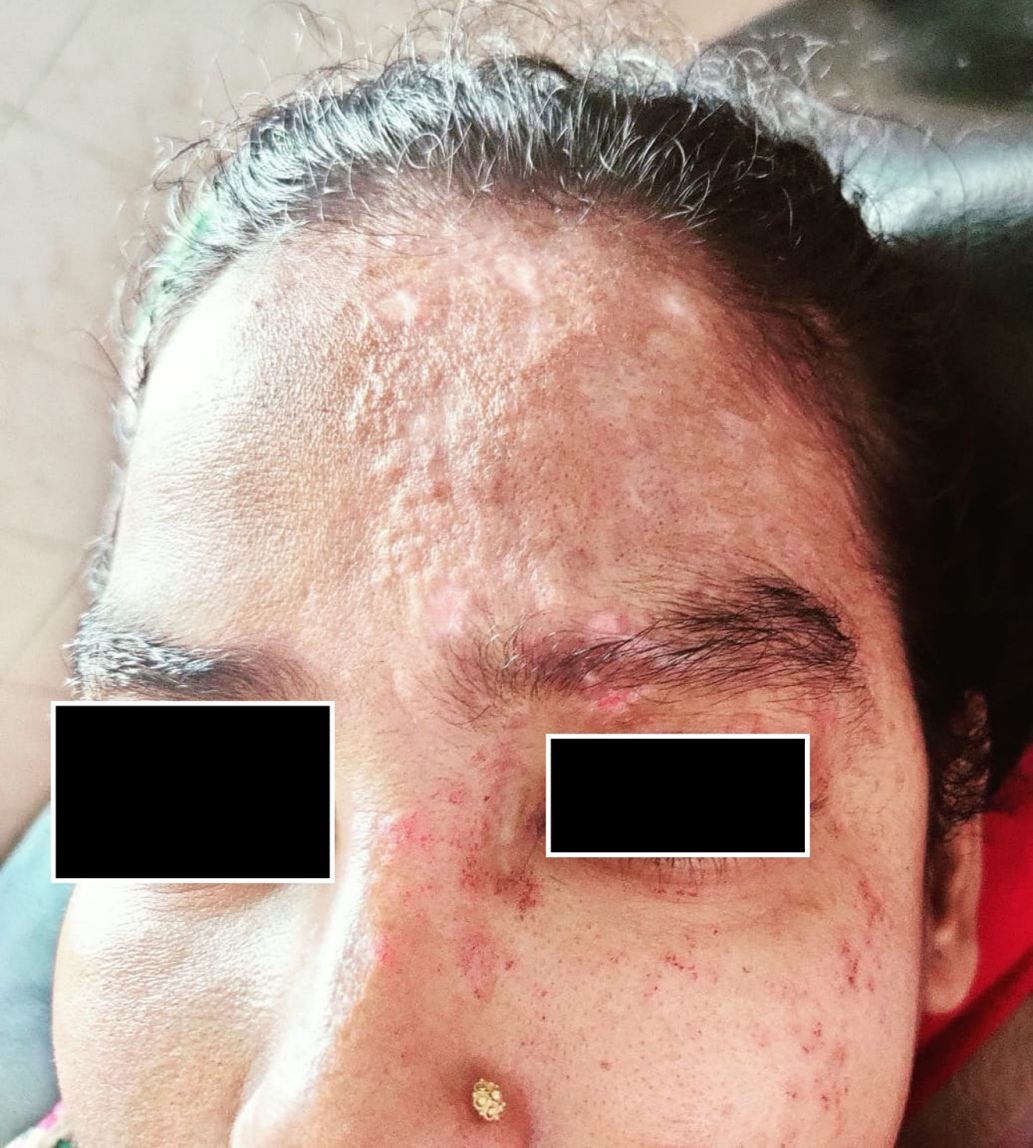
Skin lesions in their resolution phase involving the dermatomal distribution of the trigeminal nerve.

Twenty‐five days after transplant, she developed fever, which was low grade with a highest recorded temperature of 101° F. CMV‐DNA for polymerase chain reaction (PCR) was sent to rule out CMV infection. Two days later, she developed pain and vesicular eruptions involving the dermatomal distribution of the trigeminal nerve on the left (Figure 1). It was not preceded by any other prodromal symptoms. Anti‐VZV antibodies and fluid analysis from the blisters were planned, but the patient could not afford the investigations.

### 3.2 Treatment

Since she had a history of childhood chicken pox and clinically the lesion was pathognomonic of VZV infection, treatment was immediately started with oral Acyclovir 1200 mg/day, Acyclovir ointment and Ganciclovir eye gel, along with topical fusidic acid cream to prevent secondary infections.

Temporarily stopping MPS was discussed with the patient along with its pros and cons, but considering the raised Creatinine, she did not want to risk a rejection and decided to continue. Serum Tacrolimus level rose after initiation of Acyclovir and the dose was adjusted accordingly. A week later, serum CMV DNA result came back positive yielding 300 copies/mL and Acyclovir was replaced with oral Valganciclovir 450 mg/day to address both infections. Serum Creatinine rose to 3.2 mg/dL and total white blood cell count fell to 2 × 10^9^/L. MPS was temporarily stopped until count increased.

### 3.3 Outcome and follow‐up

**FIGURE 2:**
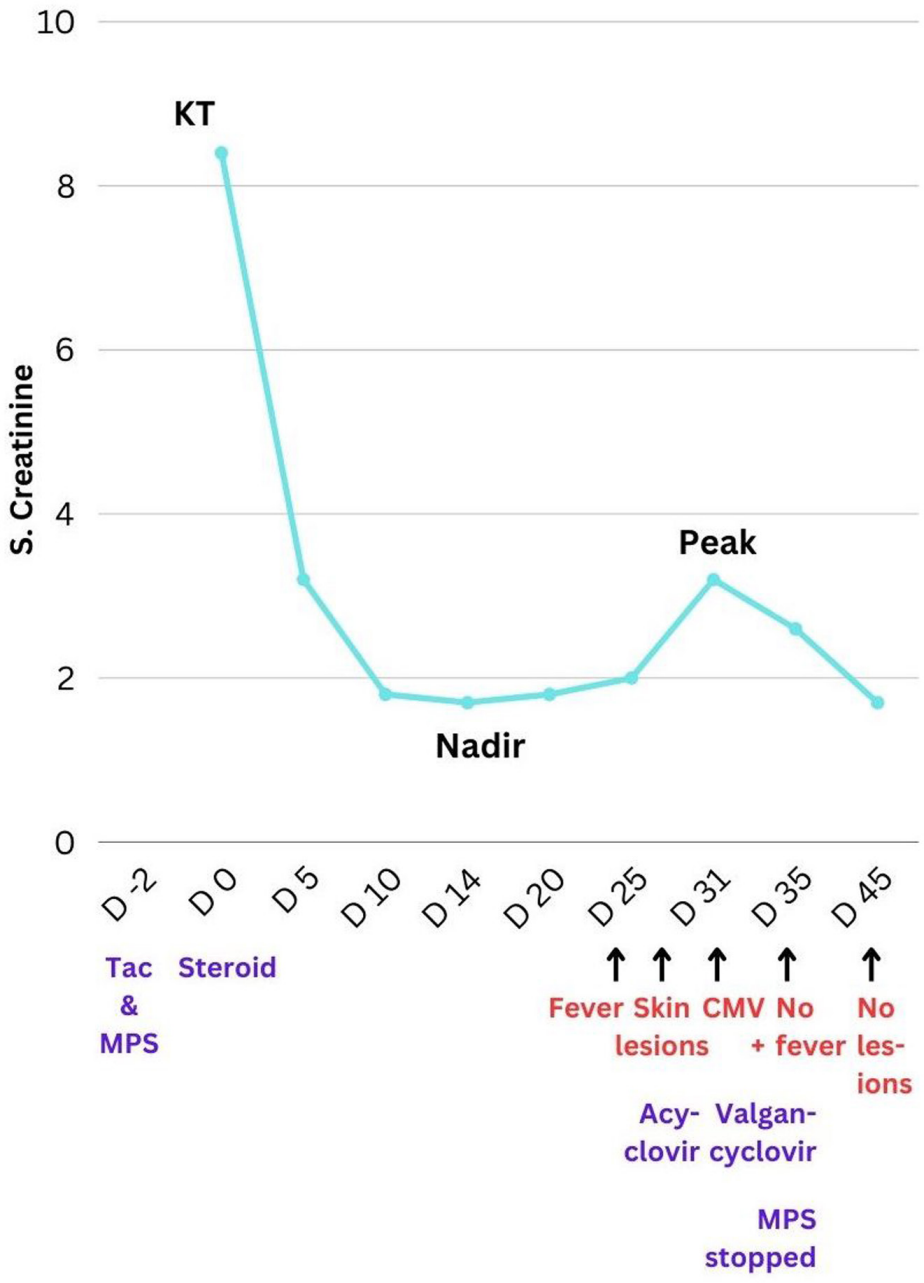
Timeline of events from during the peri‐transplant period (*x*‐axis showing time in days and *y*‐axis showing serum Creatinine level in mg/dl); KT, kidney transplant; MPS, mycophenolate sodium, Tac, tacrolimus.

The patient clinically improved with this treatment regimen. Her fever subsided and the skin lesions resolved over a period of 2 weeks. Serum creatinine came down to baseline (Figure 2). Total count also became normal. However, she developed postherpetic neuralgia, which was managed with oral pregabalin. Other follow‐up investigations were not done due to financial constraints.

## 4 DISCUSSION

VZV is a common infection after kidney transplantation. The incidence is about 28 per 1000 person‐years.^8^ The median time of onset ranges from about 6 months to 2 years.^9^ It can manifest in several ways in kidney transplant recipients—localized involving a dermatome, disseminated cutaneous, visceral etc.^10^ Usually, primary infection results in disseminated and reactivation results in dermatomal involvement.^11^ In our patient it was localized involving the Trigeminal nerve dermatome. Herpes Zoster Ophthalmicus can be vision threatening. Ophthalmological consultation was taken for our patient and vision was regularly followed up. Involvement of the facial dermatomes carries the additional risks of complications like Ramsay Hunt Syndrome, which did not occur in our case.^9^

Several risk factors have been associated with VZV infection following kidney transplantation. Although these vary among studies, increasing age of the recipient, induction therapy with depleting agents, intensification of immunosuppressive therapy suspecting rejection and seronegativity to VZV at the time of transplant are associated with increased risk.^8^, ^12^ Intensification of steroid regiment is applicable in our case.

Anti‐VZV IgM, Tzanck smear and VZV DNA are all excellent diagnostic tools for confirming VZV infection. But more often than not, feasibility becomes an issue in a resource poor setting like ours. Also, anti‐VZV IgM has low sensitivity. With the textbook dermatomal skin lesions, we decided to rely on our clinical judgment and start treatment without any delays.^13^ VZV infection needs to be treated promptly in renal transplantation patients since complications are common. Some of the complications are hepatitis, pneumonitis, pancreatitis, cerebritis, disseminated intravascular coagulation, postherpetic neuralgia (PHN) etc. Disseminated infection carries a mortality risk of 34%.^10^ In our case, being a localized disease, the patient responded well to Acyclovir and did not develop any such complication other than PHN. PHN incidence ranged from around 8% to 28% in other studies.^9^ Another complication observed in few cases was acute kidney injury (AKI).^14^ Although our patient did develop deterioration of kidney function, which later improved upon initiation of therapy, it is difficult to pick VZV infection, CMV infection or rejection as the underlying cause without a proper biopsy report.

CMV is the commonest viral infection following any solid organ transplant.^2^ Although our patient did not demonstrate any localizing evidence of CMV infection, CMV DNA detection and reduced WBC count confirmed this diagnosis. Without disseminated VZV infection and no evidence of secondary infection, it would also explain the fever. Rather than being another co‐infection, CMV may have played a more significant role in the VZV infection. CMV has an immunomodulatory effect. It affects the B cell, T cell, macrophage and phagocytic function, most notably the T cells. As in our patient, where we observed leukopenia, this can make the patient vulnerable to another infection like VZV.^5^, ^15^ The role of ongoing anti‐viral prophylaxis for CMV is less clear. Few studies have reported lower risk of VZV infection with ongoing prophylaxis, and few have found no association.^8^ CMV and VZV both being common infections in kidney transplant patients and with CMV's immunomodulatory effect, we could have expected CMV and VZV co‐infection to occur. But although multiple cases of BK virus, HHV 7 and possibly 6 coinfections were reported with CMV, there has been only one reported case of CMV nephritis and coinfection with VZV to our knowledge.^6^, ^7^

Transplantation is the best way to offer ESKD patients a normal or near‐normal life. Financial constraints, increased infection risks and lack of resources make it difficult in developing nations. Following the international guidelines in terms of diagnosis and management is often not feasible. But even with this limitations, good clinical acumen and effort can lead to a positive end result, as was a case with this patient.

## 5 CONCLUSION

Infections after kidney transplant is often difficult to diagnose, manage and cure, especially in the immediate posttransplant period. Weighing the pros and cons of altering the immunosuppressive regimen, starting antimicrobials with potential nephrotoxicity is a difficult task and needs to be tailored to individual scenarios. Our case of VZV and CMV coinfection was no different. Early diagnosis, early start of anti‐viral therapy, decision regarding continuing routine immunosuppressives while closely monitoring the patient for signs of dissemination and worsening eventually culminated in a good overall outcome for the patient's graft and her life.

## AUTHOR CONTRIBUTIONS

**Amit Bari:** Conceptualization; investigation; methodology; resources; writing – original draft; writing – review and editing. **Nura Afza Salma Begum:** Formal analysis; investigation; writing – original draft; writing – review and editing. **Farnaz Nobi:** Investigation; writing – original draft; writing – review and editing. **Harun Ur Rashid:** Investigation; supervision; writing – review and editing. **Niyoti Akhter:** Investigation; writing – original draft; writing – review and editing. **Sumona Islam:** Conceptualization; investigation; resources; supervision; writing – original draft; writing – review and editing.

## FUNDING INFORMATION

No funding.

## CONFLICT OF INTEREST STATEMENT

On behalf of all authors, I declare that there is no conflict of interest.

## CONSENT STATEMENT

Written informed consent was obtained from the patient to publish this report in accordance with the journal's patient consent policy.

## Data Availability

The data that support the findings of this case report are available and can be provided upon request.
